# Evaluation of hard palate and cleft morphology in neonates with Pierre Robin Sequence and Cleft Palate Only

**DOI:** 10.1111/ocr.12818

**Published:** 2024-05-23

**Authors:** Ines Willershausen, Nils Krautkremer, Armin Ströbel, Tariq Abu‐Tair, Friedrich Paulsen, Karin Strobel, Markus Kopp, Matthias Stefan May, Michael Uder, Franziska Krautkremer, Lina Gölz

**Affiliations:** ^1^ Department of Orthodontics and Orofacial Orthopedics Friedrich‐Alexander‐University Erlangen‐Nürnberg Erlangen Germany; ^2^ Department of Oral and Maxillofacial Surgery, School of Medicine Technische Universität München Munich Germany; ^3^ Center for Clinical Studies (CCS) Friedrich‐Alexander‐University Erlangen‐Nürnberg Erlangen Germany; ^4^ Department of Pediatric Cardiology Friedrich‐Alexander‐University Erlangen‐Nürnberg Erlangen Germany; ^5^ Institute of Functional and Clinical Anatomy Friedrich‐Alexander‐University Erlangen‐Nürnberg Erlangen Germany; ^6^ Institute of Radiology Friedrich‐Alexander‐University Erlangen‐Nürnberg Erlangen Germany

**Keywords:** cleft palate only, digital orthodontics, MRI, paediatric radiology, Pierre Robin sequence

## Abstract

**Objectives:**

This study aimed to establish a fully digital measurement protocol for standardizing the description of hard palate and cleft morphology in neonates with an isolated cleft palate (CPO) and Pierre Robin sequence (PRS).

**Materials and Methods:**

A total of 20 digitized plaster models of neonates with CPO and 20 digitized plaster models of neonates with PRS were retrospectively investigated. For the control group, the hard palate was segmented from 21 pre‐existing 1.5 T MRI datasets of neonates and exported as an STL file. The digital models were marked with predefined reference points by three raters. Distance, angular, and area measurements were performed using Blender and MeshLab.

**Results:**

Neonates with CPO (20.20 ± 2.33 mm) and PRS (21.41 ± 1.81 mm) had a significantly shorter hard palate than the control group (23.44 ± 2.24 mm) (CPO vs. control: *P* < .001; PRS vs. control: *P* = .014). Notably, neonates with PRS (33.05 ± 1.95 mm) demonstrated a significantly wider intertuberosity distance than those with CPO (30.52 ± 2.28 mm) (*P* = .012). Furthermore, there were also significant differences measured between the cleft and control groups (25.22 ± 2.50 mm) (*P* < .001).

**Conclusions:**

The data from this study demonstrate the feasibility of using MRI datasets to generate digital models of the hard palate. The presence of a cleft palate leads to pronounced adaptations of the total palatal surface area, dorsal width, and length of the hard palate. Mandibular retrognathia and altered tongue position in PRS, as opposed to CPO, might further impact palatal morphology and intertuberosity distance.

## 1 INTRODUCTION

With an incidence of 1 in 700 live births, orofacial clefts (OFCs) are the second most frequent congenital malformations after heart defects.^1^ Cleft palate only (CPO) makes up for the smallest fraction within the collective OFCs, affecting 1 in 2000 live births.^2^ From an aetiologic perspective, cleft palate can have both a syndromic background and a non‐syndromic background and occur in Pierre Robin sequence (PRS).^2^, ^3^, ^4^ PRS is a rare clinical diagnosis characterized by a hypoplastic mandible and consecutive airway obstruction due to pronounced glossoptosis.^5^, ^6^ Although cleft palate is present in most PRS cases, it is an optional, non‐defining feature of sequence.^5^ Approximately 60% of patients with PRS show a syndromic association, with Stickler syndrome being the most frequently described.^7^, ^8^ Post‐natal airway obstruction and respiratory distress are often observed in PRS due to tongue displacement and the resulting obstruction of the anterior respiratory tract.^9^, ^10^ Furthermore, adequate food intake is often challenging, and timely treatment is vital to avoid malnutrition.^11^ Pre‐natal knowledge is decisive since post‐natal care should be performed in the centre of maximum care, comprising a multidisciplinary team of paediatricians, maxillofacial surgeons, and orthodontists.^12^, ^13^ A pre‐natal ultrasound can diagnose orofacial cleft as early as 15 weeks; however, detecting isolated cleft palate and PRS specifically is challenging and unreliable.^14^ In conjunction with ultrasounds, MRI investigations, which measure the inferior facial angle, seem to aid in improving pre‐natal diagnosis.^14^, ^15^ With regard to specification of the cleft area, U‐shaped palate has been described in PRS, as opposed to V‐shaped palate in children with isolated cleft palate.^16^, ^17^ This semicircular cleft design in PRS is attributed to tongue displacement between the palatine shelves due to the posterior position of the mandibular retrognathia, mechanically hindering hard palate closure.^17^ The tongue's position results in mechanical obstruction of cleft closure, leading to a U‐shaped cleft. V‐shaped defects, on the other hand, are more likely to be associated with cleft palate only and originate from a primary failure of palatal closure.^17^ However, U‐ and V‐shaped clefts are primarily described as a visual diagnosis, with no cut‐off value concerning angular measurements of the cleft being described in the literature.^16^, ^17^ In addition to cleft morphology, the literature on neonatal palate morphology in PRS, CPO, and healthy controls is relatively scarce. This study aimed to establish a digital measurement protocol for standardizing hard palate and cleft anatomy in neonates with CPO and PRS. Furthermore, the influence of the tongue's position on intrauterine palatal growth should be evaluated by comparing both cleft groups to an age‐matched control group with physiological palatal growth. Since taking hard palate impressions of healthy neonates is not ethically defensible, the feasibility of segmenting the hard palate from pre‐existing MRI datasets was further elucidated in this study.

## 2 MATERIALS AND METHODS

**FIGURE 1:**
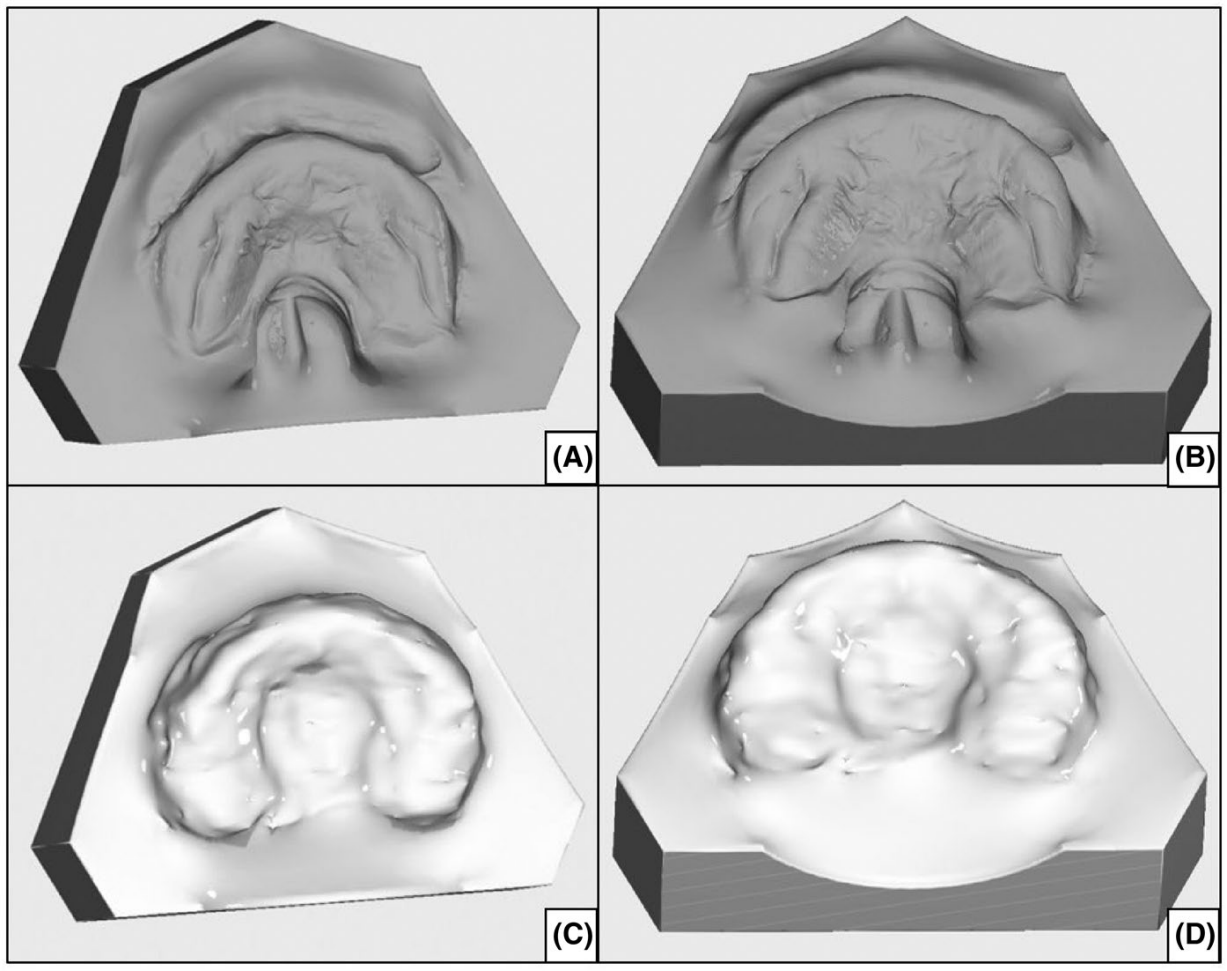
(A, B) display digitized plaster models of a neonate with PRS. (C, D) illustrate a digital model from the control group, segmented from a 1.5 T MRI dataset.

**FIGURE 2:**
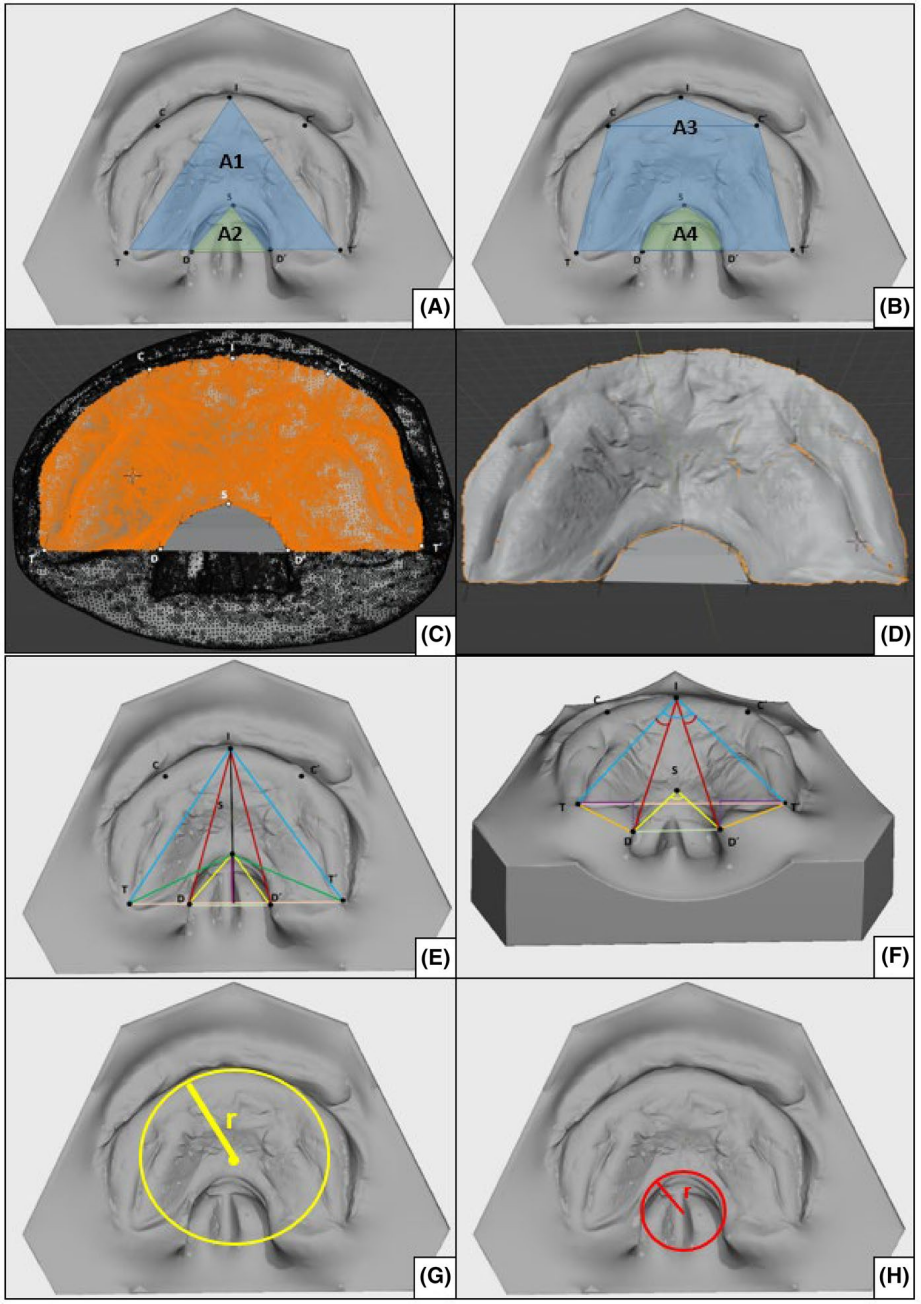
A, An illustration of the geometric area approximation of the total area (A1, triangle) and the cleft area (A2, triangle). B, An illustration of the geometric area approximation of the total area (A3, trapezoid and triangle); an illustration of the geometric area approximation of the cleft area (A4, trapezoid and triangle). C, The marked mesh area (orange) of the palatal surface and constructed cleft surface (grey) in Blender. D, The duplicated and segmented palatal and surface for export from Blender. E, The occlusal view of the digitized cast of isolated cleft palate with the predefined reference points I (incisivum), C/C′ (canine prominence right/left), T/T′ (tuber maxillae point right/left), S (foremost point of the cleft region), D/D′ (dorsal boundary of the cleft right/left), G/G′ (waypoint; half distance between C and I resp. C′ and I), and L/L′ (waypoint, half distance between D and S or D′ and S) and distances IT or IT′ (light blue), ID or ID′ (dark red), SD or SD′ (yellow), and ST or ST′ (green). F, The oblique view plotted angles ∡TIT′, ∡DSD′, ∡TID, and ∡D′IT′. G, An illustration of the hard palate's arch angle (BFCRidge). H, An illustration of the arch angel cleft (BFCCleft).

After the local institutional review board gave their consent (IRB Number: 22‐94‐Br), a total of 61 digital 3D models of neonates with CPO and PRS, as well as control groups, were retrospectively investigated. The study population consisted of 20 neonates diagnosed with non‐syndromic cleft palate only and 20 neonates with non‐syndromic PRS who received primary cleft treatment between 1981 and 2018. Plaster models of the CPO and PRS groups were scanned using a model scanner (Zirkonzahn, Gais, Italy/South Tyrol), and post‐processing was conducted using OnyxCeph (OnyxCeph3TM, Chemnitz, Germany). The final digital models were saved as STL files for further analysis in the blender suit (Stichting Blender Foundation, Amsterdam, the Netherlands) (Figure 1A,B). The control group consisted of 21 digital models segmented from T1‐weighted 1.5 Tesla MRI datasets of neonates with intact palates, which were collected between 2017 and 2022 as part of trauma or perinatal asphyxia investigations. The MRI datasets (Siemens Healthcare GmbH, MAGNETOM Sola 1.5 T: TE 2.7 ms, TR 1970 ms, flip angle 15°, field of view 250 × 250 mm, matrix 256 × 246, voxel size 1 mm; acquisition time: 4:25 min) were exported as DICOM files and segmented in 3D Slicer (3D Slicer image computing platform; 3D Slicer) using a segment editor and additional manual post‐processing (Figure 1C,D, Appendix 1)^18^ ^,^ ^19^ The methodology of segmenting the hard palate from MRI datasets is a novel approach and was, therefore, validated beforehand using MRI datasets of fully edentulous individuals, from which plaster models of maxillary and mandibular dentition were also available. Respective distance measurements for C–C′ (intercanine distance) and T–T′ (intertuberosity distance) yielded excellent agreement with an ICC >0,9 for both measurements. To avoid bias due to varying gestational age, weight, length, and head circumference, the respective z‐scores were aligned beforehand (Appendix 2). For distance and angular measurements, previously described anatomic reference points were utilized and marked in Blender^20^, ^21^ (Table 1, Figure 2A,B). Vector data of these reference points were extracted, and a Python script was utilized to calculate distances, angles, and geometric surface approximations of the overall palate and the isolated cleft region (Table 1; Figure 2).

**TABLE 1:** The anatomic reference points, distances, and angles measured in Blender.

Point		
I		Foramen incisivum point
S		Tip of the cleft region
T/T‘		Tuber maxillae
D/D‘		The dorsal border point of the cleft region
C/C‘		Canine point

Distance		
Right	Left	
IT	IT′	Distance between I and T/T′
ID	ID′	Distance between I and D/D′
SD	SD′	Distance between S and D/D′
ST	ST′	Distance between S and T/T′
TD	TD′	Distance between D/D′ and T/T′
ProjDT	ProjD′T′	Projected distance DT/D′T′ onto TT′
*Unilateral*		
ITT		Perpendicular from I onto distance TT′
SDD		Perpendicular from S onto distance DD′
TT′		Intertuberosity distance
DD′		The dorsal border of the cleft region

Angle		
Right	Left	
DIT	T′ID′	Left/right aperture angle
*Unilateral*		
TIT		Aperture angle overall palate
DSD		Aperture angle cleft region

Two differential methods were used to measure the surface area (mm^2^). An approximation of the surface area using geometric shapes (i.e., triangles and trapeziums) based on the reference points marked beforehand was further elucidated for simplification purposes (Figure 2A,B). The exact surface area of the hard palate and the cleft region (MeasAreaITT) was measured by selecting the respective areas in Blender and subsequently exporting the selected mesh (STL file) to MeshLab (Visual Computing Lab, ISTI‐CNR, Pisa, Italy) for geometric measurement (Figure 2C,D). Three different calibrated raters were used to perform measurements independently to verify the reliability of the described methodology. For intrarater reliability, one rater assessed all models a second time after 2 weeks. Distances were measured in mm, areas were measured in mm^2^, and angles were measured in degrees.

## 3 STATISTICAL ANALYSIS

A statistical analysis was conducted using R version 4.2.1. For descriptive statistics, mean and standard deviations were utilized for numerical variables, while absolute and relative frequencies were computed for nominal and ordinal variables. The two‐sample Kolmogorov‐Smirnov test was employed for numeric variables, whereas nominal and ordinal variables were compared using the chi‐squared test for independence. All the variables' intraclass correlation coefficients (ICCs) were calculated simultaneously for all three raters. The coefficient ICC (1, 2) was chosen; the absolute agreement between the judges' ratings was assessed using a two‐way ANOVA.^22^ The pooling measurements were evaluated using a pairwise ICC greater than 0.6 between raters. As a result, 18 variables of 38 were pooled across all three raters (A1, A2, A3, A4, BFCCleft, BFCRidge, ID, ID′, IS, IT, IT′, RatioA1A2, RatioA3A4, SD, SD′, SDD′, ST, and ST′), 12 variables were pooled across two raters (CC′, CleftRidge, DD′, DIT, DSD′, ITT′, ProjD′T′, ProjDT, T′D′, TD, T′ID′, and TT′), and five variables (ICC′, RatioProj, RatioSTST′, RatioTDT′D′, and TIT′) were not evaluated further since ICCs between the raters were <0.6. Three variables (MeasAreaITT, MeasAreaSDD, and RatioMeasArea) were only measured by one rater; the ICC could not be estimated, but the variables were still included in further evaluations.

### 3.1 Sample size

As this study was retrospective, the sample size was fixed at 61 neonates (20 patients per group). A two‐sample *t* test with 20 patients per group can detect an effect size d of 0.91 (a large effect according to Cohen) with a power of 80% and a significance level of 5% (software R, function power.t.test). The relationship between the effect size d and the effect size index *f* of the ANOVA can be expressed as *f* = *d*/√2 *k*, where *k* is the number of groups.^23^ When *k* = 3 groups are considered, the effect size index *f* is 0.37, a substantial effect size for the ANOVA. As this study is explorative, no corrections for multiple testing were applied.

## 4 RESULTS

### 4.1 Demographics

The retrospective study population comprised 61 neonates: 20 patients with non‐syndromic cleft palate only (*m* = 15%, n = 3; *f* = 85% n = 17), 20 patients with non‐syndromic Pierre Robin sequence (*m* = 15%, n = 3; *f* = 85% n = 17), and 21 controls (*m* = 48%, n = 10, *f* = 52% n = 11). The demographic specifications are listed in Appendix 2. No significant differences in length, weight, and head circumference were observed between the three groups, ensuring a homogenous investigation collective.

### 4.2 Angular measurements

**FIGURE 3:**
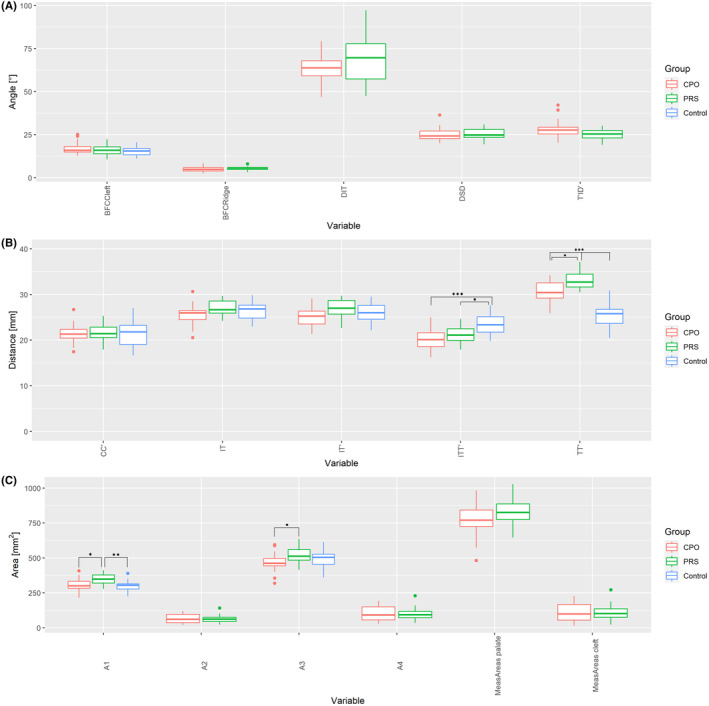
A, Angular measurements of the digitized models. BFCRidge, arch angle of the entire palate measured in all three groups. All other parameters describe the cleft area and were only measured in the CPO and PRS groups. BFCCleft, arch angle of the cleft; DSD, opening angle of the cleft; BFCCleft, arch angle of the cleft; DIT and T′ID′, cleft opening angle on the right and left sides. B, Distance measurements of the digitized models. CC′, intercanine distance; IT/IT_, distance between I and T/T′; TT′, posterior palatal width; ITT_, sagittal length of the hard palate. All distances are measured in mm. *P < 0.05, ***P < 0.001. C, Area measurements of the digitized models. A1 and A3, area approximation of the total hard palate; A2 and A4, area approximation of the cleft area; MeasAreapalate, exact surface of the entire palate; MeasAreacleft, exact surface of the cleft region. *P < .05, **P < .01.

Angular measurements were employed to further describe the anatomy of the cleft region and the hard palate. The angle DSD, which describes the opening angle of the cleft area, tended to be wider in the PRS group (69.48° ± 14.34°) than in the CPO group (63.59° ± 8.20°); however, only a trend towards significance could be observed here (*P* = 0.081). The clefts' opening angle was further investigated on the left and right sides of the cleft (DIT and T′ID′). However, no difference concerning laterality was observed here. The arch angle of the cleft (BFCCleft) was greater in the PRS group (5.33 ± 1.24°) as opposed to the CPO group (4.93 ± 1.53°); however, this was not significant. The control group (15.43 ± 2.63°) exhibited smaller values for the arch angle of the entire palate (BFCRidge) compared to the CPO (16.92 ± 3.49°) and PRS (16.04 ± 3.15°) groups; however, these differences were also non‐significant (Figures 2E‐H and 3A).

### 4.3 Distance measurements

Distance measurements were used to depict differences in hard palate anatomy in the CPO, PRS, and control groups. Although no significant differences were observed between the CPO PRS groups in terms of the anterior transversal width of the hard palate (CC′), the underlying diagnosis significantly impacted the intertuberosity distance (TT′). A significant difference concerning posterior palatal width was observed between both the PRS group (33.05 ± 1.95 mm) and the CPO group (30.52 ± 2.28 mm) (*P* = 0.012). Highly significant differences were further observed between both investigation groups and the control group (25.22 ± 2.50 mm) (*P* < 0.001). In addition to the transversal plane, the sagittal length of the hard palate (ITT_) was also influenced by the group affiliation. While no significant differences were observed between both investigation groups (CPO (20.20 ± 2.33 mm) vs. PRS (21.41 ± 1.81 mm)), the hard palate of neonates with CPO and PRS was significantly shorter than those of the control group (23.44 ± 2.24 mm) (CPO, *P* < 0.001; PRS, *P* = 0.014) (Figures 2E,F and 3B).

### 4.4 Area measurements

When comparing the three groups concerning areas A1 and A3, which represent an approximation of the total area of the palate, marked differences were observed (Figure 2A,B). Neonates with PRS (346.69 ± 36.96 mm^2^) showed a significantly (*P* = 0.034) greater A1 surface area than those with CPO (305.81 ± 45.31 mm^2^), with the smallest A1 surface area found in the control group (295.59 ± 41.4 mm^2^). Comparable results were obtained for the A3 area, with the PRS group presenting significantly higher values than the CPO group (*P* = 0.034) (Figure 3C, Table 2). No significant differences between the PRS and CPO groups related to the approximation of the isolated cleft area (A2) were observed. Furthermore, no differences between the PRS and CPO groups regarding A2 and A4, which represent surface approximations of the cleft area, were observed. Both cleft groups were investigated regarding the exact surface of the cleft region (MeasAreacleft); no difference could be observed (PRS 109.11 ± 56.61 mm^2^; CPO 107.91 ± 68.32 mm^2^). The exact surface area of the entire hard palate (MeasAreapalate) tended to be larger in the PRS group (830.23 ± 99.11 mm^2^) than in the CPO group (777.77 ± 121.70 mm^2^); however, these differences were non‐significant.

**TABLE 2:** Overview of all measured variables.

	CPO (n = 20)	PRS (n = 20)	Control (n = 21)	*P*
A1	305.81 + −45.31 (20)	346.69 + −36.96 (20)	295.59 + −41.41 (21)	<.001
A2	66.25 + −33.89 (20)	63.51 + −28.29 (20)		.892
A3	469.09 + −68.12 (20)	514.65 + −60.01 (20)	485.62 + −71.81 (21)	.134
A4	102.19 + −54.63 (20)	98.83 + −45.66 (20)		.957
MeasAreapalate	777.77 + −121.70 (20)	830.23 + −99.11 (20)		.137
MeasAreacleft	107.91 + −68.32 (20)	109.11 + −56.61 (20)		.850
CC′	21.44 + −2.18 (20)	21.66 + −1.85 (20)	21.67 + −2.82 (21)	.881
DD′	11.70 + −3.66 (20)	12.69 + −2.31 (20)		.449
ID	23.40 + −2.71 (20)	24.48 + −1.72 (20)		.279
ID′	23.35 + −2.72 (20)	24.36 + −1.89 (20)		.317
IS	14.55 + −4.42 (20)	16.14 + −3.49 (20)		.330
IT	25.47 + −2.20 (20)	26.99 + −1.60 (20)	26.51 + −2.00 (21)	.064
IT′	25.22 + −2.17 (20)	26.90 + −1.98 (20)	25.98 + −2.03 (21)	.045
ITT′	20.20 + −2.33 (20)	21.41 + −1.81 (20)	23.44 + −2.24 (21)	<.001
ProjD′T′	10.08 + −1.47 (20)	10.38 + −1.16 (20)		.449
ProjDT	9.05 + −1.07 (20)	10.33 + −1.11 (20)		.002
T′D′	11.97 + −1.79 (20)	11.50 + −1.27 (20)		.245
TD	10.93 + −1.43 (20)	11.71 + −1.24 (20)		.088
TT′	30.52 + −2.28 (20)	33.05 + −1.95 (20)	25.22 + −2.50 (21)	<.001
BFCCleft	4.93 + −1.53 (20)	5.33 + −1.24 (20)		.317
BFCRidge	16.92 + −3.49 (20)	16.04 + −3.15 (20)	15.43 + −2.63 (21)	.457
DIT	25.34 + −4.00 (20)	25.41 + −3.17 (20)		.665
DSD	63.59 + −8.20 (20)	69.48 + −14.34 (20)		.144
T′ID′	28.36 + −5.35 (20)	25.11 + −3.16 (20)		.040

*Note*: Measurements of the cleft area are only possible in the CPO and PRS groups. If data were available, *P*‐values were used to compare all three groups; otherwise, CPO and PRS were compared.

## 5 DISCUSSION

The results of this study illustrate that the presence of a cleft palate is associated with significant three‐dimensional changes in the hard palate in neonates with both PRS and CPO. In this study, neonates with CPO and PRS showed a significantly shorter hard palate than the control group (CPO 20.20 ± 2.33 mm vs. PRS 21.41 ± 1.81 mm vs. control group 23.44 ± 2.24 mm) (Table 2). However, no significant differences were observed between both cleft groups, supporting the hypothesis that the sole presence of cleft palate is associated with sagittal growth inhibition. Although the literature on hard palate morphology in healthy neonates is extremely scarce, our measurements align with previously published data that describe palatal growth and symmetry in healthy neonates.^24^, ^25^ In our collective of healthy neonates, the intertuberosity distance measured 25.22 ± 2.50 mm, which is comparable with the results of Bruggink et al..^25^ Concerning intertuberosity distance, highly significant differences were observed between the three investigation groups (*P* < .001). In the two cleft groups, neonates with PRS presented with a significantly wider intertuberosity distance than those with CPO (PRS 33.05 ± 1.95 mm vs. CPO 30.52 ± 2.28 mm) (*P* = .012). Although the literature on the width of the posterior palate in neonates with isolated cleft palate is quite rare, it has been investigated in complete and incomplete unilateral clefts.^21^, ^26^, ^27^ Pronounced differences between complete and incomplete unilateral clefts could be observed, with the complete cleft group yielding significantly higher values than the incomplete group.^21^ Although Neuschulz et al. provided no birth percentiles concerning weight and height, the values described for the incomplete cleft group are comparable to those in our PRS group. The hypothesis that altered tongue position leads to morphological variations in the hard palate is supported by these data.^21^ While the intertuberosity distance between the three investigated groups varied significantly, with the highest values found in the PRS group, interestingly enough, no significant differences concerning dorsal width of the cleft region (DD′) could be observed between the PRS and CPO groups. In addition, DT and D′T′, describing dorsal width of the left and right palatal segments, presented no significant differences. Evaluating these results in a three‐dimensional context, the changes mentioned above in intertuberosity distance may occur due to the rotation of palatal segments around a sagittal axis. In the literature, cleft palate associated with PRS has been described as U‐shaped, whereas cleft palate associated with CPO is V‐shaped, depending on their formation mechanism.^17^ For an objective assessment of cleft shape, the opening angle of the cleft region ($∡{\mathrm{DSD}}^{\prime }$), as well as the radius of the best fit circle calculated for cleft and palatal ridge (BFCCleft and BFCRidge), was compared in our study (Figure 3G,H). However, there were no significant differences; rather, there was a tendency towards higher values in PRS. Therefore, the concept of V‐ and U‐shaped clefts could not be verified in our study. The values for both the exact and area approximations were highest in the PRS group. These observations may be partially attributed to the inclusion of TT′ in the measurements, suggesting that the variations in size are primarily a result of this factor.

This study presents some limitations regarding the unequal gender distribution in the CPO group, the limited sample size due to the prevalence of CPO, and the retrospective design. The results of this study suggest that the presence of a cleft palate is associated with a pronounced morphological alteration of intrauterine hard palate growth in patients with both PRS and CPO, thereby showing a reduction in sagittal and an increase in posterior transversal growth. Mandibular microretrognathia and glossoptosis in PRS, as opposed to CPO, seem to further impact palatal morphology and intertuberosity distance. Our findings underline the importance of an early diagnosis and timely treatment of PRS and CPO to enable adequate nutrition and physiological development of the hard palate in patients with cleft palate.

## AUTHOR CONTRIBUTIONS

I.W. contributed to conception, design, data acquisition, and interpretation and drafted and critically revised the manuscript. N.K. contributed to conception and design and critically revised the manuscript. A.S. conducted the statistical analysis and critically revised the manuscript. T.A‐T. contributed to conception and data acquisition and critically revised the manuscript. F.P. contributed to conception and critically revised the manuscript. K.S. treated the patients and critically revised the manuscript. M.K., M.S.M., and M.U. provided the MRI datasets and critically revised the manuscript. F.K. contributed to conception, design, data acquisition, and interpretation and critically revised the manuscript. L.G. contributed to conception, design, and interpretation and critically revised the manuscript.

## CONFLICT OF INTEREST STATEMENT

M.K., M.U., and M.S.M. are members of the speaker's bureau of Siemens Healthcare GmbH. All other authors declare no potential conflicts of interest with respect to the research, authorship, and/or publication of this article.

## ETHICAL INFORMATION

The ethics committee of the Friedrich‐Alexander‐University Erlangen‐Nürnberg, Erlangen, Germany, approved this study (IRB Number: 22‐94‐Br).

## Data Availability

The data are not publicly available due to privacy or ethical restrictions. The data of this study are available from the corresponding author upon reasonable request.

## APPENDIX 2

### Demographic specifications of the three different investigation groups.

**Table:**

	Cleft palate only [n = 20]	Pierre Robin [n = 20]	Control group [n = 21]	*P*
Length [cm]	49.99 + −3.58 (16)	50.27 + −2.05 (15)	50.35 + −3.47 (20)	.881
Percentile length	39.67 + −35.39 (15)	33.67 + −27.20 (15)	47.65 + −27.75 (20)	.472
Z‐Score length	−0.55 + −1.49 (15)	−0.54 + −0.86 (15)	−0.18 + −1.12 (20)	.477
Weight [g]	3045.59 + −852.09 (17)	3125.62 + −414.76 (16)	3096.90 + −518.36 (21)	.979
Percentile weight	38.44 + −39.55 (16)	33.53 + −23.85 (15)	37.70 + −26.25 (20)	.814
Z‐Score weight	−0.51 + −1.62 (16)	−0.54 + −0.83 (15)	−0.44 + −0.93 (20)	.780
Head circumference	34.28 + −1.95 (16)	34.34 + −1.26 (12)	33.75 + −2.12 (20)	.594
Sex				
Female	15% (3)	55% (11)	48% (10)	.022
Male	85% (17)	45% (9)	52% (11)	

*Note*: The variables length, weight, and head circumference showed no significant differences between the groups, ensuring a structurally identical collective.
